# Blockchain-Based Authentication and Trust Management Mechanism for Smart Cities

**DOI:** 10.3390/s22072604

**Published:** 2022-03-29

**Authors:** Muhammad Asif, Zeeshan Aziz, Maaz Bin Ahmad, Adnan Khalid, Hammad Abdul Waris, Asfandyar Gilani

**Affiliations:** 1 Department of Computer Science, Lahore Garrison University, Lahore 54000, Pakistan; hammadzahid2254@gmail.com (H.A.W.); s.gilani@lgu.edu.pk (A.G.); 2 School of Science, Engineering and Environment, University of Salford, The Crescent, Salford M5 4WT, UK; z.aziz@salford.ac.uk; 3 Karachi Institute of Economics and Technology, College of Computing and Information Sciences, Karachi 75190, Pakistan; maaz@kiet.edu.pk; 4 Department of Computer Science, Government College (GC) University, Lahore 54000, Pakistan; adnan.khalid@gcu.edu.pk

**Keywords:** information security, blockchain, internet of things, smart cities, authentication, authorization, Ethereum

## Abstract

Security has always been the main concern for the internet of things (IoT)-based systems. Blockchain, with its decentralized and distributed design, prevents the risks of the existing centralized methodologies. Conventional security and privacy architectures are inapplicable in the spectrum of IoT due to its resource constraints. To overcome this problem, this paper presents a Blockchain-based security mechanism that enables secure authorized access to smart city resources. The presented mechanism comprises the ACE (Authentication and Authorization for Constrained Environments) framework-based authorization Blockchain and the OSCAR (Object Security Architecture for the Internet of Things) object security model. The Blockchain lays out a flexible and trustless authorization mechanism, while OSCAR makes use of a public ledger to structure multicast groups for authorized clients. Moreover, a meteor-based application is developed to provide a user-friendly interface for heterogeneous technologies belonging to the smart city. The users would be able to interact with and control their smart city resources such as traffic lights, smart electric meters, surveillance cameras, etc., through this application. To evaluate the performance and feasibility of the proposed mechanism, the authorization Blockchain is implemented on top of the Ethereum network. The authentication mechanism is developed in the node.js server and a smart city is simulated with the help of Raspberry Pi B+. Furthermore, mocha and chai frameworks are used to assess the performance of the system. Experimental results reveal that the authentication response time is less than 100 ms even if the average hand-shaking time increases with the number of clients.

## 1. Introduction

The internet of things (IoT) refers to a system of interrelated computing devices, physical and virtual objects, digital and mechanical machines, actuators, home appliances, etc., that enables these things to connect, interact, and transfer data over the internet [1]. It allows Internet Protocol (IP) enabled objects to be sensed or controlled through the network and integrate these devices with existing internet infrastructure [2]. IoT is the most emerging point of discussion these days [3]. All the bigwigs of Information Technology (IT) are considering it as a future option. Every coming day brings a new IoT implementation to enable products and applications. The most prominent IoT applications are smart homes, smart cities, smart grid, smart retail, smart farming, smart supply chain, wearable’s, connected car, connected health, industrial internet, etc. [4]. The deployment of IoT-based applications brings a revolution in the life of human beings.

Even though there is a dramatic increment in the deployment of IoT enabled products and applications, IoT still has many addressable security challenges [5]. People still believe that security issues are the basic hurdle in the evolution of IoT-based solutions. The main issue with IoT is that there is no proposed generalized security system that would be able to counter different types of security attacks because each gadget taking part in the system has its security risks, limitations, and vulnerabilities.

**Figure 1:**
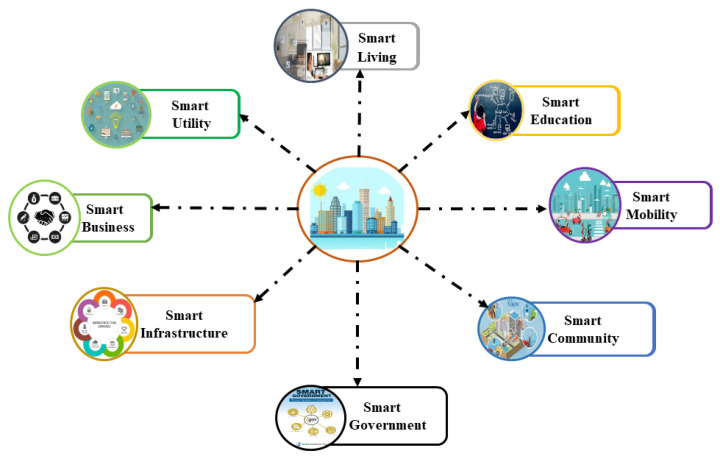
Smart cities main features.

A smart city is an urban area in which diverse kinds of electronic sensors are used to gather data from devices, assets, and citizens [6,7]. The collected data are analyzed and processed to monitor and manage power plants, waste management, hospital, schools, libraries, traffic, transportation systems, information systems, and other community services [8,9,10]. Figure 1 depicts the main features of smart cities. As the model for smart cities involves the use of public data, many security attacks and privacy risks are possible at multiple levels of the smart city architecture. So, it is the key requirement for such an architecture that security and privacy must be ensured.

Blockchain is the foundation of Bitcoin and was firstly proposed in 2008-9 [11,12,13,14]. At present, all tycoons of IT are considering it as a security option because of its versatility and trustless way of authorization. Blockchain is a distributed ledger technology (DLT). It offers a consensus-based validation process. Moreover, it also provides peer-to-peer networking that removed the requirement for any centralized mechanism for operating in terms of security and privacy [15]. Blockchain records all the transactions in the ledger which are available to all nodes, and transactions are performed in a decentralized manner. Blockchain-based applications are rapidly growing in different fields such as IoT, reputation system, financial services, etc.

So, the most feasible solution for security problems in IoT is the Blockchain. Let us take an example of CCTV cameras operating in a smart city to clarify this concept. Government organization deploys these cameras at several public points for security surveillance. Here, the government organization serves as the resource owner and CCTV cameras are considered as a single resource of the smart city. Security department officials are the clients of this resource. To authenticate the clients, the government organization publishes a smart contract on the Blockchain. To prove the authenticity of the client, the smart contract may need some kind of proof. This proof is necessary to ensure whether a particular client belongs to a security department or not. This proof then becomes the part of the transaction’s data field that is sent to the smart contract. On the execution of the smart contract, a token is generated for the client. The account address of the client is referred by the token. A token has a life-time field and is associated with a particular resource. There can be many smart contracts offered by a single resource owner that may be deployed on the same resource server on Blockchain. Each contract has different parameters and there could be many smart contracts for the different resources of smart city; each one of these may have different parameters and attributes. In such a context, the smart contract has the parameters which would be used to identify and authenticate the legitimacy of the security institution’s personnel. If they are a legitimate one, then tokens would be assigned to them, otherwise the request would be abandoned by the smart contract.

Blockchain is recently used to provide security and privacy to IoT devices and related topologies [16,17,18,19,20,21,22]. IoTChain is a Blockchain-based architecture to provide an end-to-end (E2E) solution for secure authorized access to IoT resources [23,24]. It is a combination of the Object Security Architecture for the Internet of Things (OSCAR) and the ACE (Authentication and Authorization for Constrained Environments) authorization framework of the Internet Engineering Task Force (IETF). Preventing unauthorized access to resources of the smart city is a serious concern. A strong temper-proof authentication mechanism is required to accomplish it. Moreover, there is also a need for a user interface to control and monitor resources of the smart city. The proposed mechanism should be comprehensive in nature. All the implementation details must be clear to the reader and transparent to the end-users.

Conventional security and privacy architectures are not suitable in the spectrum of IoT due to its resource constraints [25]. Moreover, most of the IoT devices are not designed to meet the basic security and privacy parameters which produce the security, confidentiality, privacy, authentication, data integrity, and access control issues. Blockchain is gaining significance in the field of IoT by enhancing security and empowering the incorporation of increasing devices into the ecosystem [26,27,28].

In order to provide such a solution, a Blockchain-based mechanism is presented in this work for secure authorization of smart city resources. It consists of the ACE framework-based authorization Blockchain and the OSCAR object security model. The Blockchain offers a flexible and trustless authorization mechanism. On the other hand, OSCAR makes use of a public ledger to establish multicast groups for authorized clients. Moreover, a meteor-based application is developed to provide a user-friendly interface for heterogeneous technologies belonging to the smart city to interact and control the smart city’s resources. To evaluate the performance and feasibility of the proposed mechanism, the implementation of authorization Blockchain is made on the top of the Ethereum network. The security mechanism is developed in node.js server and a smart city is simulated with the help of Raspberry Pi B+. Furthermore, mocha and chai frameworks are used to access the performance of different components of the mechanism. Following are the main contributions of this paper:

- Designed an efficient Blockchain-based authentication and trust management mechanism for smart cities;
- A practical implementation of a Blockchain-based security mechanism for secure authorization of smart city resources;
- Developed a user-friendly hybrid application to interact and control heterogeneous IoT resources.

It would help to provide the following security goals:

- To mutually authenticate clients and servers and establish trust among them so that only legitimate users may access the system and the servers may become validated by the clients;
- To secure the key exchange mechanism among clients and servers;
- To authorize the users so that a resource can only be accessed by the users authorized for that resource;
- To provide monitoring and control of the smart city resources to the manager/admin of the system.

The rest of the paper is organized as follows: Related work is given in Section 2. The overview of Blockchain technology is given in Section 3. Section 4 describes the proposed solution. The experimental analysis and discussion is made in Section 5. Finally, a conclusion is drawn in Section 6.

## 2. Related Work

Many researchers proposed different architectures for the use of Blockchain in multiple domains. Samaniego and Deters [29] presented software-defined IoT devices in combination with permissioned Blockchain for enabling the edge hosts to IoT services. How to implement software-defined IoT devices as a smart contract is an interesting research question. Kravitz and Cooper [30] presented a theoretical methodology for establishing an ecosystem using permissioned Blockchain. Identity management is a built-in part of it for devices and users. It is an interesting framework, but no implementation has been conducted yet.

Dorri et al. [31] proposed a lightweight implementation of Blockchain for resource- and energy-constrained devices of IoT. Proof-of-work protocol used in most of the IoT consumes a lot of energy, so it is a major research problem to overtake it. Ethereum Casper protocol was recently released which should provide a possibility to create more robust IoT-Blockchain systems. IOTA [32] is a third-generation decentralized cryptocurrency. It is an application of IoT having advanced features for keeping records and processing transactions. Its implementation is not yet available. IoTeX [33] is a decentralized network for IoT. It claims fast consensus and built-in privacy-preserving techniques that enable the physical and virtual things to securely exchange information and value at a global scale. So, it is the coming future technology based on the IoT-Blockchain platform having more privacy, security, and scalability for developing new IoT-based applications.

Watson IoT platform [34] enables you to add chosen IoT data into a private Blockchain through a built-in function. It makes use of the IBM cloud, so it is helpful to use in a corporate environment in terms of scalability and quick adaptation to the changing business needs. Ambrosus [35] is a Blockchain-based IoT network. It was developed for the field of the pharmaceutical and food departments. It enables IoT sensors to talk to Blockchain. It assures data integrity for products and improves the standard and tracking of items throughout the supply chain process. Waltonchain [36] opens up the epoch of value internet of things (VIOT). It alters label, trade, organization, and network. It is useful in RFID technology and mobile phones. It uses asymmetric encryption algorithms and uses a combination of Blockchain and IoT [19].

Origin trail [37] is the protocol of Blockchain-powered data exchange for interconnected devices. It is a decentralized protocol running on a network of public nodes and enables Blockchain-supported data sharing in the supply chain. Atonomi [38] provides security protocol and enables IoT devices to have security and reliability in data for eCommerce applications. It is bringing enormous IoT devices on Blockchain’s immutable ledger [16]. This architecture is only specific to E-commerce devices and is not extended to smart cities. IoTChain [23] uses OSCAR and ACE schemes. It is used to provide an E2E solution for secure authorization access to the internet of things resources. It makes the process of resource access secure, scalable, robust, and flexible.

Yup et al. [39] presented a Blockchain-based technique for intelligent healthcare. They developed a data gateway and data access control to ensure the user’s privacy. Liang et al. [40] utilized Blockchain technology to develop a system to share healthcare records through a mobile application. They suggested a user-centric technique to provide secure access control and privacy with the help of channel formation methodology. Jiang et al. [41] presented a Blockchain-based medical information exchange system. They developed an on-chain and off-chain verification mechanism to secure the system storage. Fan et al. [42] suggested Blockchain-based patient medical data along with improved consensus protocol to enhance the overall security and privacy of the system. Tanwar et al. [43] targeted healthcare 4.0 applications and proposed an electronic healthcare record system based on Blockchain. They presented a patient-centric scheme to provide symmetric key cryptographic access control to various healthcare providers. They also used the concept of chaincode to develop a permission-based electronic health recode sharing system.

Habibzadeh et al. [44] performed a survey on security and policy issues for the systems deployed in the smart city environment. The authors showed that applications developed for smart cities are vulnerable and technology and government policies must work together to eliminate these vulnerabilities. They visualized smart city as a multi-level system having certain security level. Technical and policy challenges and future directions for a secure smart city are encapsulated in this work. Khan et al. [45] presented a Blockchain-based system to verify data of CCTV cameras in smart cities. The system helps to identify fake video recordings, enabling the investigation officers to find the tempered videos. Blockchain is used to avoid the single point of failure issue of such systems and also ensures that video recordings are authentic and not modified. Hyperledger fabric is used to implement and evaluate the proposed system. Results show that data are distributed among different nodes and once a transaction is performed, the system becomes temper-proof. Only view rights are granted to the users.

Malik et al. [46] presented a framework for analyzing the patterns of users’ physical activity in a small city environment. These patterns may help to authenticate a user passively. Several machine learning algorithms are exploited to validate the identity of users by using three different datasets. The authors claimed that the proposed system provided better results than the existing systems. Meshram et al. [47] presented an authentication protocol for the smart cities environment. The proposed protocol is based on smart card and user password-based authentication and uses extended chaotic map. Some of the key features of the proposed protocol are insider attacks detection, detection of wrong password, stolen smart card detection, cancelation of lost smart cards, mutual authentication, and protection against client’s impersonation attacks. Multiple hypotheses are given and tested under the proposed protocol which shows its robustness. Esposito et al. [48] exploited Blockchain for authentication and authorization issues in smart cities applications. The authors discussed the need of security-related information management through a decentralized way. For this purpose, they integrated FIWARE with Blockchain-based access control and identity management. The comparison of the proposed system with the one using databases shows the superiority of it.

Ibba et al. [49] performed a bibliometric review based on article co-citation analysis and keyword co-occurrence network. They highlighted the challenges and discussed trends related to the deployment of Blockchain in smart cities. Suciu et al. [50] presented Tangle and Blockchain technologies and their main attributes to perform a detailed comparison of their techniques. Rejeb et al. [51] presented a Blockchain-based solution for sensors data storage and management. They applied SCRUM methodology to make Blockchain-based software due to its adaptive, flexible, and iterative nature.

In short, there is a need for a practical framework that can be deployed as such to provide security for smart cities. The heterogeneity aspect and complexity of the implementation must be transparent to the end-users. It should not only provide security but should also be scalable, robust, and flexible.

## 3. Overview of Blockchain Technology

In this era, Blockchain and smart ledger technologies are the leading research domains after the dawn of cryptocurrencies such as Ethereum and bitcoin. In Blockchain, data are stored and shared in a distributed, immutable, trusted fashion but are not editable. There is no need for intermediaries and centralized dependency to examine transactions [52,53]. The transparent nature of the Blockchain offers a low complexity procedure to retrieve the ledger-based transactions over networks. Blockchain consists of different services and approaches such as hash cryptography, mining, immutable ledger, consensus protocol, and distributed P2P Networking. Following is the detail of these methods and services:

- A Blockchain exploits hash-based encryption algorithms for the addition or updating of transactions to the ledger. The main characteristics that belong to hashing techniques are the avalanche effect, deterministic, one-way cryptography, must withstand collisions, and faster computation. Blockchain uses the SHA-256 hashing algorithm.
- Mining: It is the process of securing and verifying transactions. It demands high computing speed to attain and acquire the reward. It requires miners to add transaction data to the ledger. In the ledgers, blocks are connected and develop a chain that is secured by Blockchain miners.
- Immutable ledger: In a Blockchain network, all transactions are recorded. An immutable ledger means recorded transactions cannot be altered or tampered with.
- Consensus protocol: It is a procedure through which all the Blockchain nodes reach a common consensus regarding the present state of the distributed ledger. It helps in achieving reliability and establishing trust between unknown nodes in the Blockchain network.
- Distributed P2P Networking: To distribute and update the data at the nodes of the Blockchain network, all transactions are broadcasted over the network.

**Figure 2:**
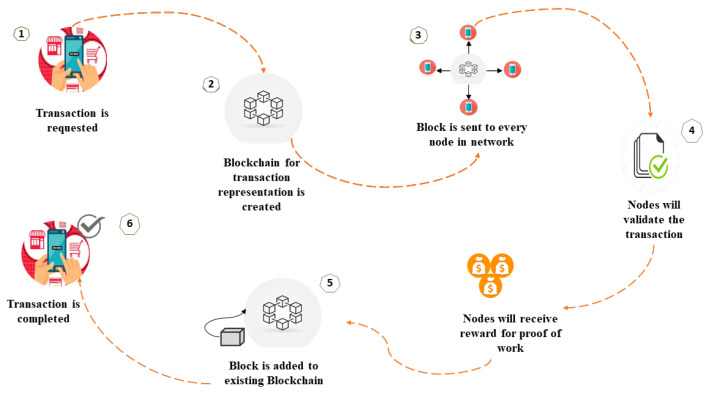
Blockchain transaction process.

Blockchain technology stores records in unaltered form in a distributed network. The addition or updating of transactions is made through the creation of new hash values. Blockchain technology has several advantages, e.g., decentralization, security, immutability, and transparency, that give it an edge over other existing technologies. Figure 2 describes the transaction process of Blockchain.

### Ethereum Blockchain Architecture

**Figure 3:**
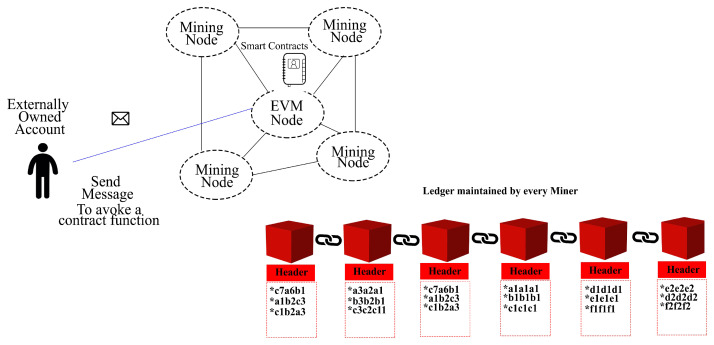
Ethereum Blockchain architecture.

Ethereum ledger consists of blocks, transactions, and consensus. The architecture comprises of Ethereum virtual machine (EVM), transactions, minor, consensus, accounts, smart contract, ethers, and gas. Figure 3 illustrates the Ethereum-oriented blockchain architecture.

A Blockchain network comprises numerous nodes. Some nodes are miners, while the rest of the nodes do not mine but facilitate smart contract execution. These are known as EVMs. These nodes are connected to establish the network and utilize peer-to-peer protocol for communication purposes. All miner nodes contain an instance of a ledger. The ledger keeps all blocks belonging to the chain. The synchronization among miners is made on an ongoing basis to ensure consistency.

**Figure 4:**
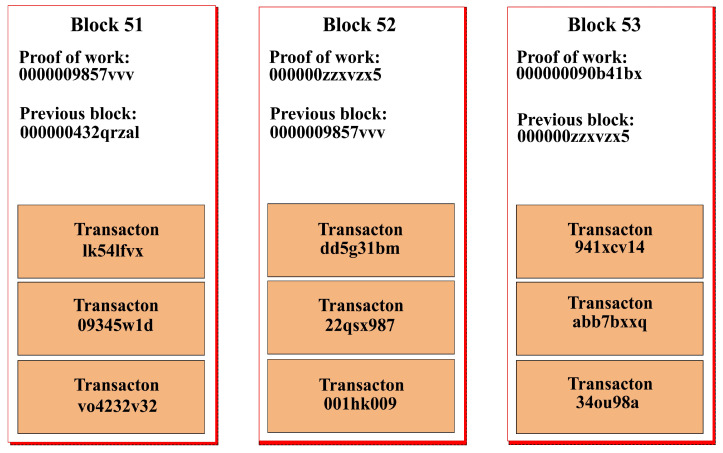
Parent–child relationship among blocks in Blockchain.

In Ethereum Blockchain, there exists a parent–child relationship among blocks. This relationship is one to one. In this way, a chain is established. Figure 4 explains the parent–child relationship.

**Figure 5:**
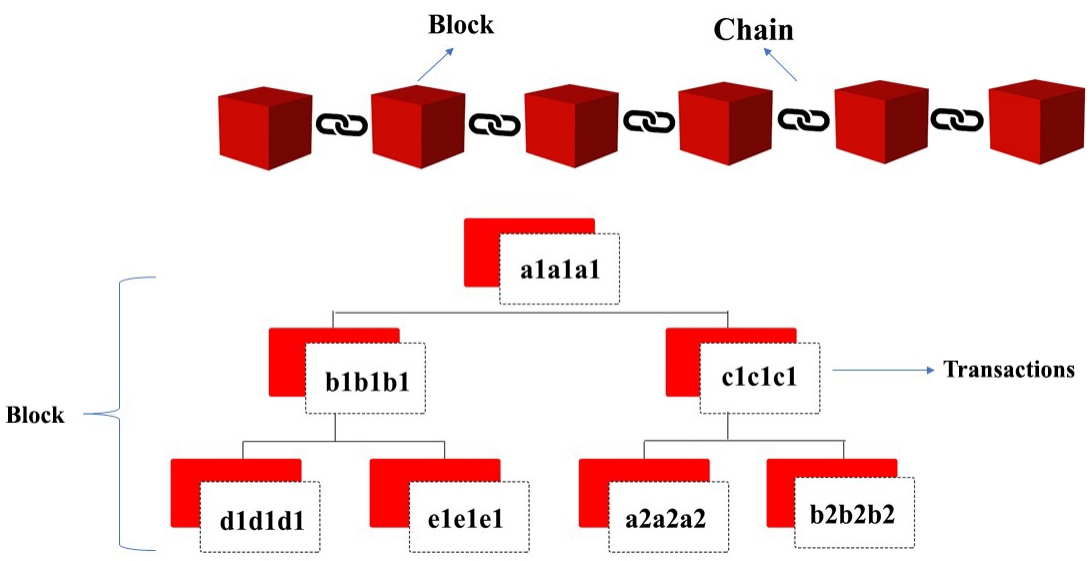
Relationship between transactions and blocks in Blockchain.

Blocks keep a record of the transactions. How many transactions a block can contain is determined by the upper gas limit. Each transaction consumes a certain amount of gas. So, when the quota of the gas for a certain block is reached, a new transaction becomes part of the next block. These hashed transactions are saved in the blocks. The hashes of two transactions are combined to produce a new hash. This mechanism keeps on running until there is a single hash from all transactions belonging to the block. The resultant single hash for the block is known as Transaction Merkle root hash and is saved in the blocks’ header. This makes the chain immutable. Figure 5 shows the relationship between transactions and blocks.

**Figure 6:**
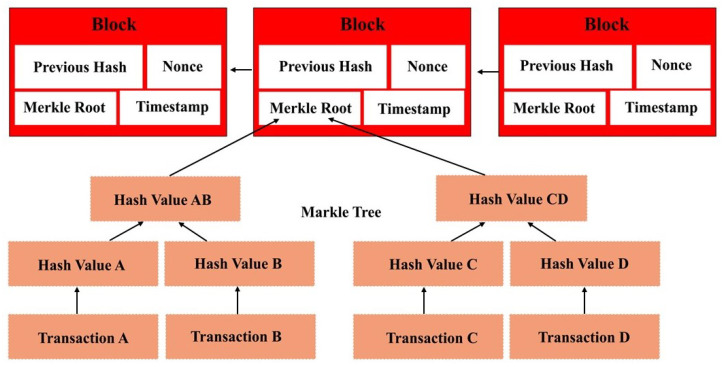
Block header and its contents.

All miners at some points gather all transactions from the pool. The miner inserts these transactions in a new block provided that these transactions are not already present in the block. A timestamp and nonce are also added to the block header. Figure 6 shows the block header and its contents.

## 4. Proposed Solution

**Figure 7:**
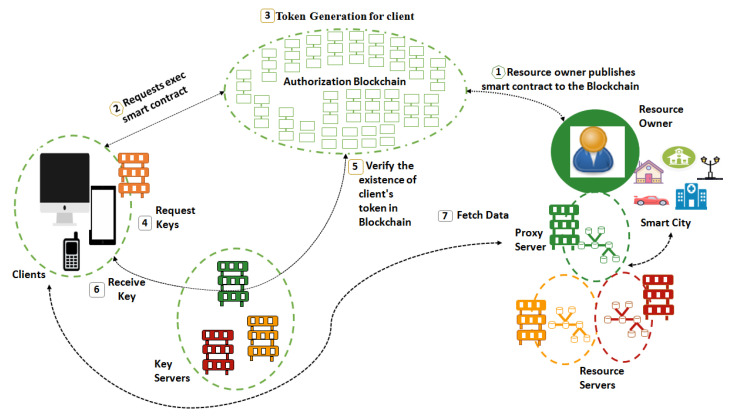
Block diagram of the proposed solution.

The proposed Blockchain-based architecture to ensure security and authorization for accessing smart city resources is shown in Figure 7. It represents the main components of the architecture and illustrates the steps toward authorized access of smart city resources. To clarify confusion related to taxonomy and working of distinct entities, the nomenclature as specified by the Internet Engineering Task Force (IETF) is as follows:

- Authorization Servers: These are used to generate access tokens.
- Resource servers: These generate/store protected resources.
- Proxy Servers: These store protected resources and act as access interface for clients.
- Resource Publishers/Owners: These are the original owners of resource servers and their corresponding resources.
- Key Servers: These are used to generate keys for encryption/decryption of the resources.
- Client/User application: Third party that requests access to the protected resources.

### 4.1. Authorization Blockchain

Generally, a Blockchain may be conceived as a log having persistency and whose records are saved in timestamped blocks. Transactions are contained by blocks. Each block is recognized by its cryptographic hash value, and it mentions the hash of the previous block. The Blockchain is maintained by the nodes that have the copy of the last n blocks (at least).

The authorization Blockchain has key servers, authorization servers, and clients as its nodes. All of these nodes do not need to store complete Blockchain and take part in the consensus protocol. There are some full nodes (e.g., authorization servers and key servers). These store the complete information of the Blockchain. Only authorization servers behave as miners. Resource owners set up the key servers who are responsible for the keys-related material of the resource servers. All other nodes are identified by a unique Blockchain address which is a pair of asymmetric keys. A smart contract is created to describe access rights by the resource owner; access tokens are then automatically generated by it. Transactions are performed to interact with smart contracts or among clients and resource owners. These transactions are signed by the private key of the respective nodes. These transactions are then broadcasted to the Blockchain network. The authorization servers (miners) verify the transactions and store those in blocks. Sealing of these blocks is performed by some consensus protocol and then these are appended to the Blockchain. The key servers and resource servers must have valid certificates to authenticate themselves. A client requests a key server to provide a decryption key for their desired resources. The client’s authorization is verified by the key server by checking the smart contract storage of Blockchain. If the client is verified, then it obtains the required key through which it can download and decrypt the desired resource.

In this work, the Ethereum [54] Blockchain network is used as an authorization Blockchain for smart city resources. In Ethereum Blockchain, customers and contractors’ records (accounts and information) are stored in timestamped blocks. It is a publicly available permissionless Blockchain for decentralized applications. Following is the rationale for using Ethereum Blockchain:

- One of the prominent features of Ethereum is that it is programmable. All the contracts and agreements are part of the code enabling transactions to be executed automatically. With the help of its smart contracts, users can exchange anything having value, e.g., money, property, etc. These smart contracts can also invoke other contracts and may have lots of conditions and supported formats.
- Ethereum is not only confined to cryptocurrency transactions but any transactions related to supply chains, energy grids, real estate, government registries, etc.
- It is proven that Ethereum is robust in nature against security attacks.
- It is open, flexible, and supports both permissionless and permissioned implementations.
- An Ethereum network can support up to hundreds of nodes and millions of users, a number much larger than its competitors.
- Ethereum consortia are not dependent on some single vendor, rather it is interoperable.

Because of Ethereum’s large ledger size, each transaction for any action/task which is written or verified from the ledger takes too much time and resources, e.g., huge gas consumption. Because of the large ledger size, e.g., 80 GB, it is not feasible to perform the development of real-time tasks on a public Blockchain until or unless the system fully matures and passes through all testing frameworks.

**Figure 8:**
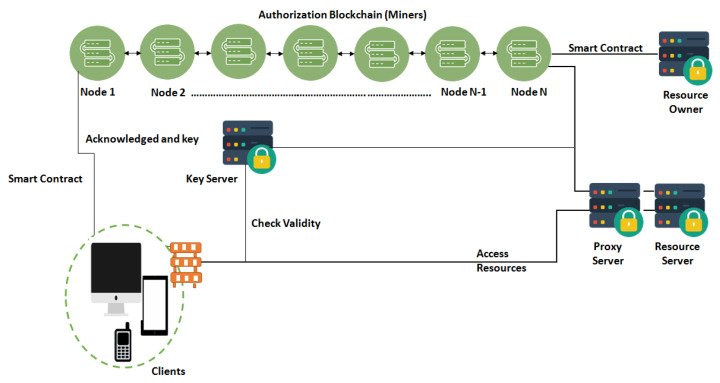
Blockchain network.

**Figure 9:**
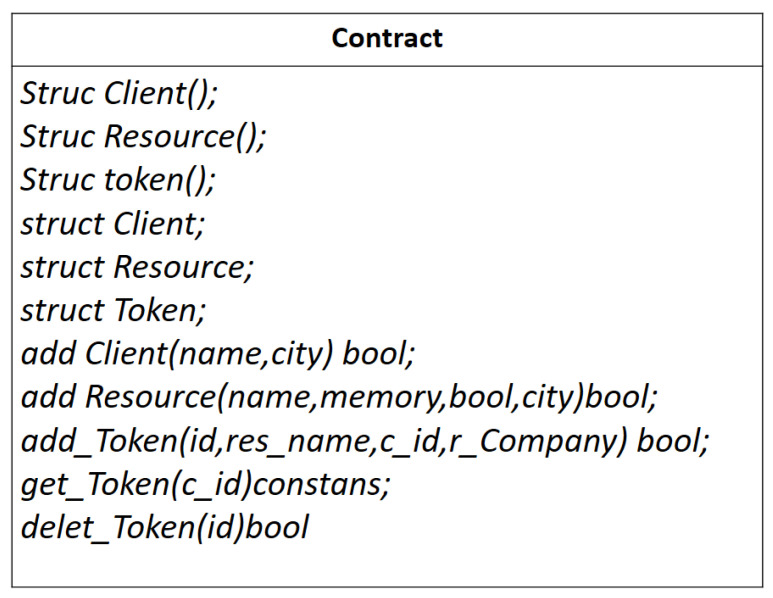
Smart contract deployed on the Blockchain.

In this work, Ganache [55] local Ethereum Blockchain is used to deploy smart contracts and migrations without bulky ledger and gas consumptions. Ganache provides ten accounts for the users. Here, the users would be either resource owners or clients. Figure 8 shows the test-net for the proof of concept. Out of these ten accounts, one is allocated to a resource server, proxy server, key server, and resource owner, each. To deploy the smart contract on the Blockchain, the resource owner connects to a full node. The smart contracts are shown in Figure 9.

Clients can start interacting with smart contracts by invoking the public functions of it. An access token is created for a client by the function addToken(). This token is saved in the persistent memory of the contract. It contains both the client and resource server addresses. When a client sends requests to a key server for a decryption key, the TTL value of the access token is checked by the key server. To revoke the access rights of the client earlier, the deleteToken() function can be used. The smart contract can be activated by any entity in the Blockchain network.

More complex contracts might need some modifiers that control access to certain functions or to check if a particular condition is satisfied before function execution by the EVM, e.g., did this client pay for the access token?

### 4.2. Resource Servers

The protected resources of smart cities are stored and generated by resource servers. In the case of smart cities, traffic lights, security surveillance cameras, power plants, water supply network components, etc., are the resources. In this work, the resource server is developed on Raspberry Pi B+ [56].

### 4.3. Resource Owners/Publishers

Resource owners are the legal owners of the resource servers and their generated resources. In the case of smart cities, a resource owner is a government institution or controlling authority.

### 4.4. Key Servers

The necessary keys to encrypt and decrypt the resources are generated by key servers. When the user meets the contract requirements, a key would be generated for a specific time for using the resources. In this work, the key server is developed on the Core i7 machine with 4GB RAM, Sony Vaio CPU 2.6 GHz, and Ubuntu 16. 04.1 operating system. The key server has a full node status in the Blockchain.

### 4.5. Simulation/Creation of Smart City

**Figure 10:**
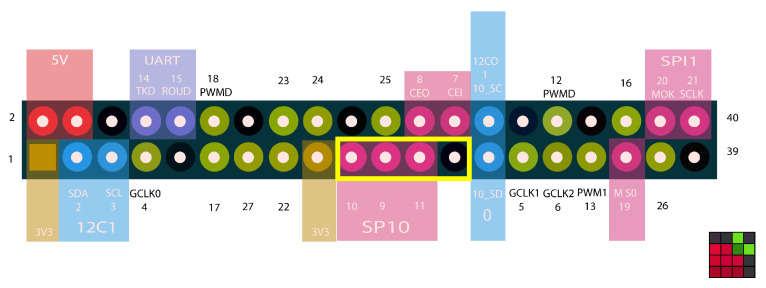
Raspberry Pi utilized GPIO pins.

The smart city utilizes different types of electronic sensors to acquire information and then use this information to control and manage resources efficiently. In this work, Raspberry Pi B+ is used for the simulation of a smart city. Two main functionalities of smart cities, i.e., traffic lights and security surveillance cameras, are simulated. In Figure 10, general purpose input pins (enclosed in the yellow rectangle) are controlled through tokens generated by the smart contract and output pins are connected with the physical smart city’s objects. To control the physical devices of the smart city, the node.js framework and git repository are installed on Raspberry Pi B+.

### 4.6. Integration of Smart City with Authorization Blockchain

To connect the smart city with authorization Blockchain, the truffle framework [55] is used. It makes the integration of smart contracts, binary management assets, contract migrations, and deployment easier.

#### 4.6.1. Smart Contract Writing

**Figure 11:**
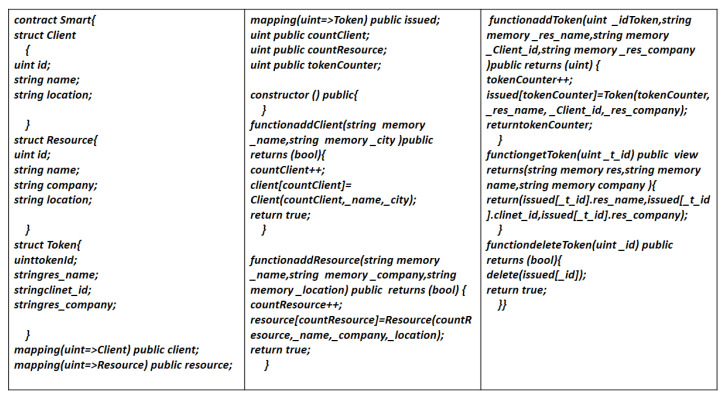
Sample smart contracts.

Smart contracts are a part of Ethereum Blockchain. Ethereum invokes it whenever a user is validated to have a sufficient transaction fee. After validation, resources are allocated to the stack holder. These are basically the applications that run over Ethereum virtual machines. Ethereum virtual machine (EVM) executes it in byte-code or a string of 0s and 1s readable and interpretable by the network. Solidity [57] is an object-oriented high-level programming language used for Ethereum-based contract writing. The sample contracts are given in Figure 11.

#### 4.6.2. Migrations

After the contract writing and module development, the migration process is performed. In the migration process, all modules such as Raspberry Pi integration with node.js [58] and smart contract integration at the js level are performed. Whenever the application is launched, the migration process executes first.

#### 4.6.3. Access Control for Blockchain at Application/Browser Level (Metamask)

To access the Ganache Blockchain, Remote Procedural Calls (RPC) are used. RPC is time taking, so to speed up the development process, metamask [59] is used which gives us Ethereum Blockchain access at the browser level. The main advantage of metamask is that developers do not need any type of RPC calls over the internet for performing any test action. To deploy proposed systems in smart cities/homes, an obvious shift is required from Ganache metamask testing Blockchain to the geth Ethereum ledger.

#### 4.6.4. Data Fetching from Smart Contract

Data fetching is performed to present the contract data (name, location, resource name, resource price, resource provider, etc.) at the application level for publishing resources, for the insertion of resources and users. In this work, Web3.js [60] is used for data fetching and insertion. Web3.js is a collection of libraries that allows the user to interact with the local and remote Ethereum using HTTP RPC.

### 4.7. User Application

**Figure 12:**
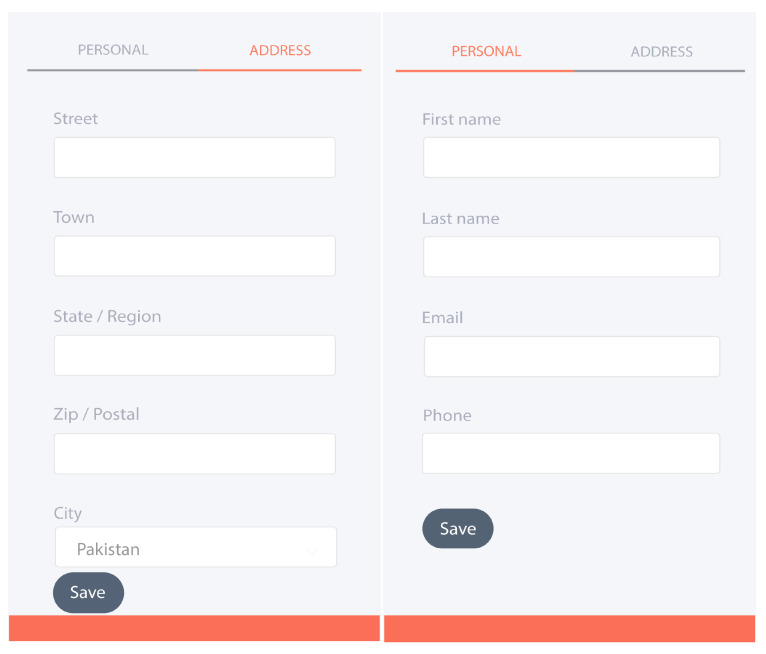
User information.

To interact and control IoTchain-based secure smart city resources, an application is required. In this work, a hybrid application is developed which can be used for mobiles (Android, iOS, Windows, Blackberry) and computers. This application is developed in Meteor.js [61] framework. To use the proposed application, the user has to perform the following steps:

- Create an Ethereum Blockchain wallet and acquire the public and private keys for wallet authentication.
- Connect wallet to metamask by providing public and private keys. The metamask provides a 14-word memic which can be used for the retrieval of the wallet in case of keys stolen.
- Open the application that asks the user to provide the necessary information as shown in Figure 12. The information provided at the time of startup is used for contract and resources provision.

### 4.8. Overall Working Flow

The following is the working flow of the proposed system and is shown in Figure 7.

- The resource owner/provider (government institutions) creates a smart contract to publish it to the Blockchain.
- The client that wants to acquire the protected resources sends a transaction to the particular smart contract address. The transaction is broadcast to all the Blockchain network nodes. The process of validation will be started, and miners will take part in it.
- If the client meets certain requirements of the contract, then an access token will be generated. The access token describes the specific access rights (start time, expiry time, location, resource name) for the protected resources.
- After the consensus, when the transaction is confirmed, smart contracts will be executed.
- The contract transaction will be added to the contract and a token will be generated which will be the basic key for further processing.
- The client then requests the key server for the necessary decryption keys to decrypt the resources.
- The key server has a replica of the token. For the access token, the key server queries the internal storage of the responsible smart contract.
- The key server generates a challenge response based on the client address given in the token to prove the authenticity of the client. This challenge can be solved by only the legitimate client that triggered the smart contract.
- The client receives a personal key and takes part in the self-healing group key distribution process after completing the challenge.
- Finally, the encrypted resources can be downloaded by client proxy or resource server. Both of these servers offer RESTful CoAP API which enables GET, POST, and PUT resources using Uniform Resource Identifier (URI).

It is to be noted that no authentication is necessary at this point because only an authorized client can obtain decryption cryptographic keys. In the case where the protected resources are acquired directly from the resource servers, a symmetric key would be used to secure the resources. On the other hand, when the proxy server is used to obtain the resources, an asymmetric signature would be used to protect the integrity of the resources. In both cases, necessary keys are provided by the key servers.

## 5. Experimental Analysis and Discussion

This section presents the performance evaluation of the main components of the proposed framework, discusses the security aspects and technical challenges, and highlights the limitations.

### 5.1. Evaluation of Key Server and Resource Server

To record the performance of different components of the Blockchain-based security mechanism, different types of tools and technologies (mocha framework and chai assertion library) are used. Mocha is a JS-based framework used for testing each module and measuring its performance. To GET, POST, or PUT data over the servers, mocha uses an assertion library named chai. To request data from a specified resource, GET method is used. For sending data to a server in order to create/update a resource, PUT method is used.

**Figure 13:**
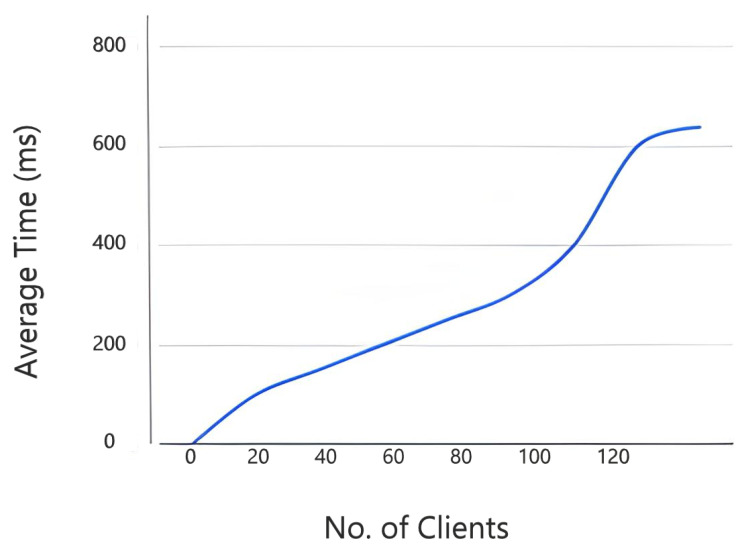
Average time to accomplish the DTLS handshake.

The key server is executed on Ubuntu 16.4 LTS. Figure 13 shows the average time required to accomplish the hand-shake process. It is observed that the average hand-shaking time increases with the increase in the number of clients. For 60 clients, average hand-shaking time is 200 ms which doubled for 120 clients. The machine used as the client is equipped with an Intel Core i7-4600M CPU @ 2.60 GHz (4 virtual cores), 4 GB RAM.

**Figure 14:**
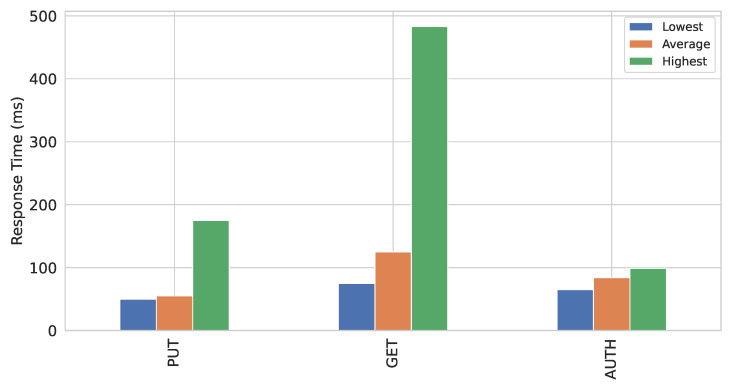
Performance of the resource server.

In this work, the resource server is deployed on Raspberry Pi B+ and is developed in C language. Figure 14 demonstrates the response time in milliseconds (ms) for a PUT and GET request from a client to a resource server. Moreover, it shows the time required to complete the DTLS handshake between a resource server and the key server which is indicated by AUTH. It illustrates the lowest, highest, and average response time for each request. It can be noted that lowest, average, and highest response time for PUT method are 50, 55, and 175 ms, respectively. Similarly, for GET method, lowest, average, and highest response time are 75, 125, and 483 ms, respectively. The lowest, average, and highest response time are 65, 84, and 99 ms, respectively, for AUTH.

**Figure 15:**
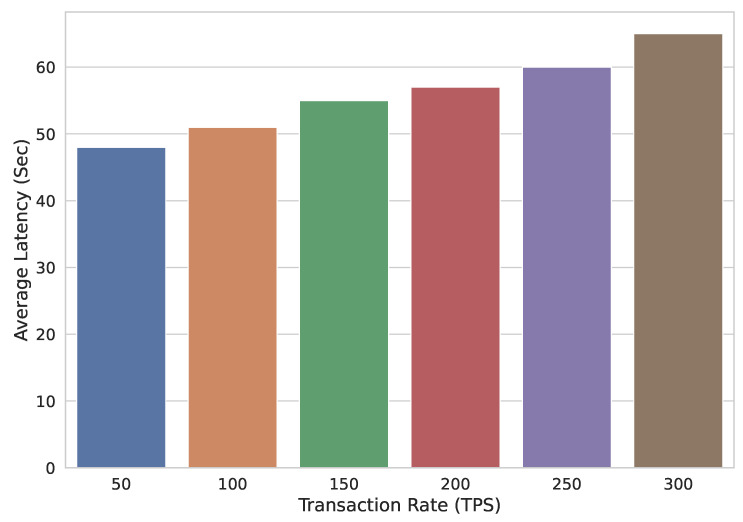
Average latency with varying transaction rate.

To further test the efficacy of the proposed system, several experiments were performed by varying different parameters, e.g., transaction rate, block size, etc. First of all, the transaction rate is varied. Transaction rate is mentioned by $T_{R}$. The value of $T_{R}$ is varied between 50–300 transactions/sec and average latency $LT_{AVG}$ is computed against each $T_{R}$ value. Figure 15 shows the transaction commit time of this experiment. It is evident that as the transaction rate is increased, $LT_{AVG}$ also increases.

**Figure 16:**
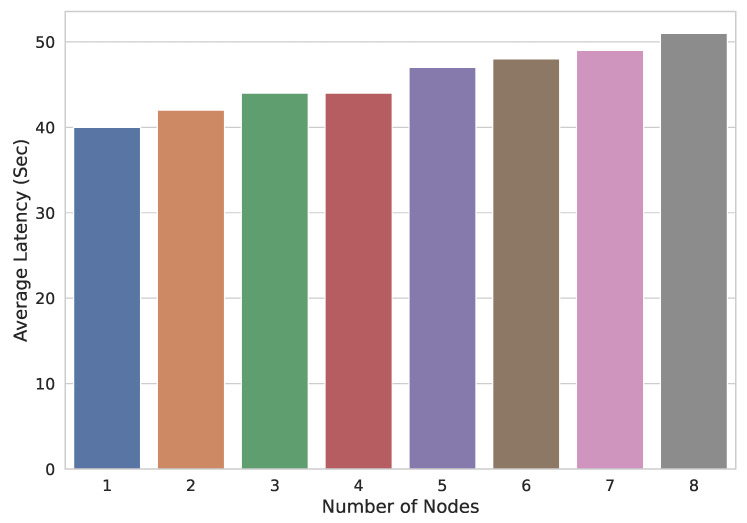
Transaction average latencies with varying number of nodes.

In the second experiment, $T_{R}$ is kept constant, i.e., 150, and the number of nodes performing these transactions keeps increasing from 1 to 8. The transaction average is shown in Figure 16. It is reflected from the results that as the number of nodes taking part in transactions increases, $LT_{AVG}$ also increases. It is lowest when the single node is performing the 150 transactions per second and is highest when 8 nodes are sharing these 150 transactions.

**Figure 17:**
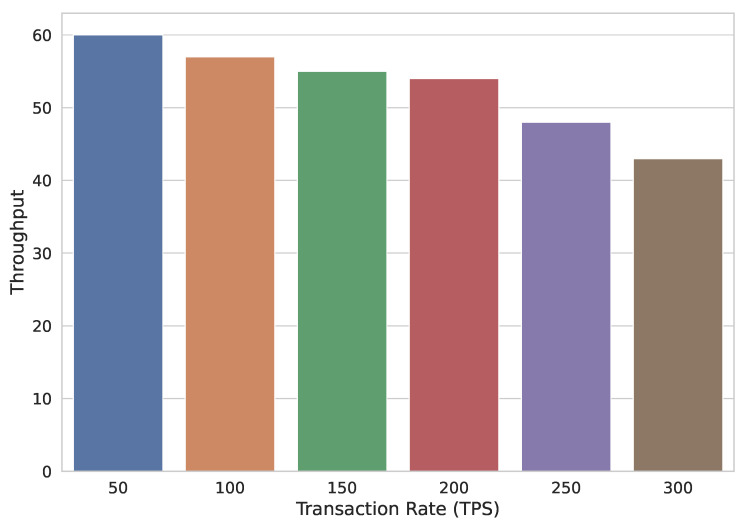
Transaction throughput with varying transaction rate.

Figure 17 shows that as the number of nodes taking part in transactions increases, the transaction throughput decreases.

**Figure 18:**
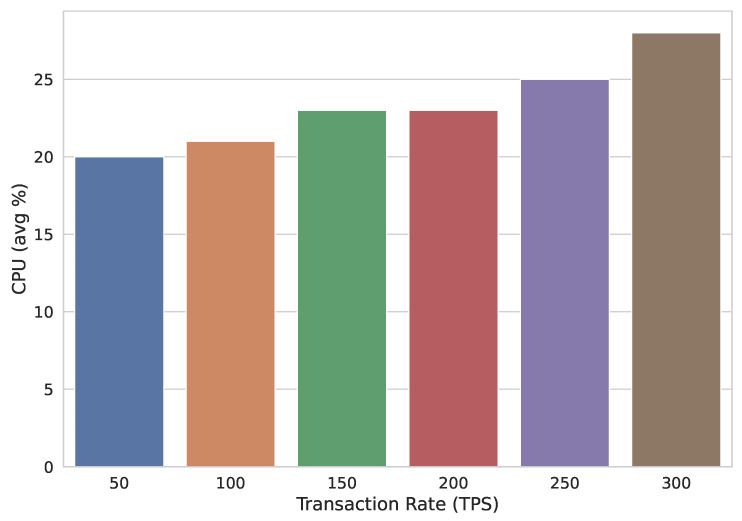
CPU usage with varying transaction rate.

Another observation regarding CPU usage is recorded when $T_{R}$ is changed. It is shown in Figure 18 that CPU utilization keeps on increasing as the $T_{R}$ is increased, keeping the block size constant.

**Figure 19:**
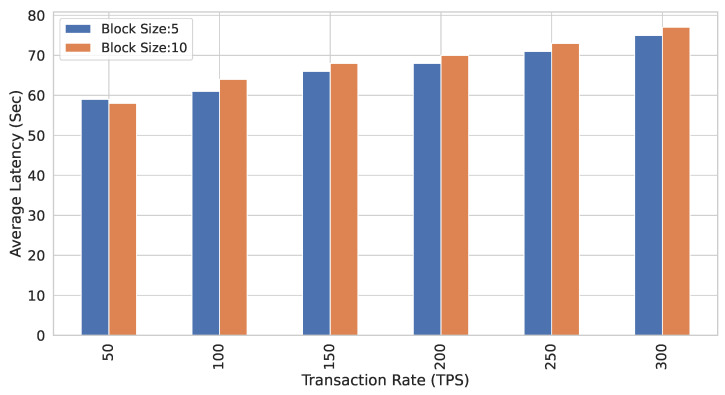
Average latency with varying transaction rate for different block sizes.

In all the above experiments, transaction size and block size are kept constant, i.e., 2 transactions/block. All the transactions are performed in the read mode. A new set of observations are recorded by varying the block sizes and finding the $LT_{AVG}$ for varying $T_{R}$. Figure 19 shows the results for this configuration. First, the block size is kept as 5, and $LT_{AVG}$ values are calculated by varying $T_{R}$. After that, the block size is increased to 10 and $LT_{AVG}$ values are recorded. It can be noted that block size 10 has a slightly lower latency than block size 5. The mode of transactions is read in all of these experiments.

In a nutshell, it is observed that low $T_{R}$ on small block sizes may improve the performance of a Blockchain system. Similarly, high $T_{R}$ on larger block sizes also improves the performance of the system.

### 5.2. Smart City Resource Publishing and Controlling through User Application

#### 5.2.1. Publishing Resource

**Figure 20:**
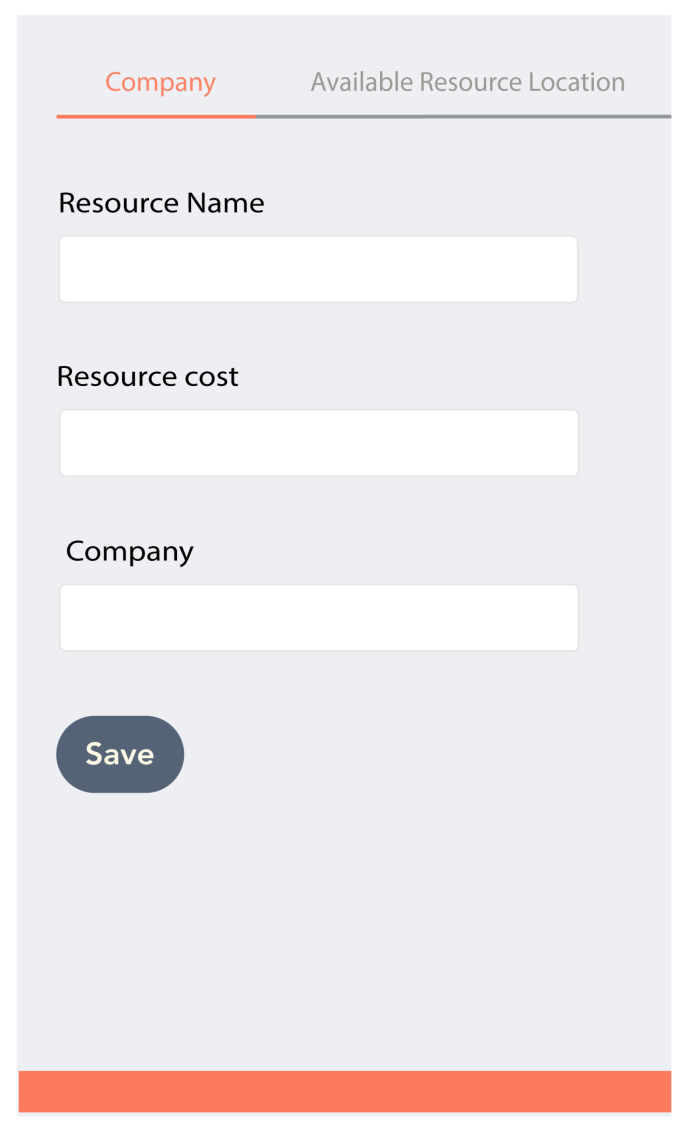
Resource publishing.

The resource provider can add or publish the smart city resource (CCTV camera, traffic lights, electricity meter, etc.) contracts to the Blockchain through user application. Let us consider a scenario; a smart city management authority has deployed smart CCTV cameras and street lights. The authority publishes a smart contract on the Blockchain in order to authorize the clients. Some kind of proof, i.e., the client lives at a particular address, may be required by the smart contracts. This proof is included in the transaction’s data field which is then sent to the smart contract. The contract generates a token for the client on execution. The token references the address of the client (i.e., a public key). The access token also has a lifetime field and describes which resources are accessible. A resource owner has the option to deploy many smart contracts for the same resource server on the Blockchain. Every contract is different in the sense that it takes dissimilar input parameters and produces tokens with different privileges. Figure 20 exhibits the page that is used for publishing the resource. Three fields are there, i.e., recourse name, resource cost, and company.

#### 5.2.2. Accessing and Controlling of Resources

**Figure 21:**
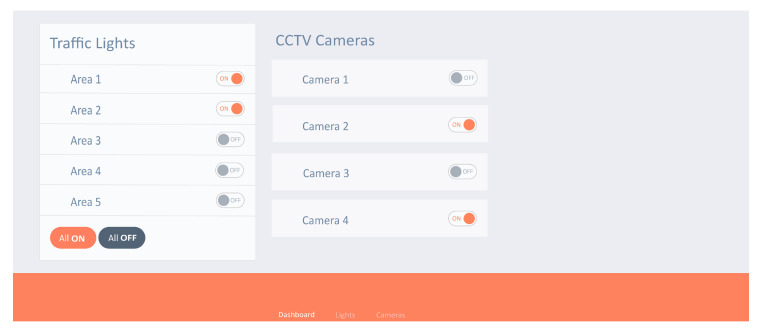
Client dashboard.

The client will be able to see and manage the available resources in its area to whom that is authenticated. Figure 21 shows the client dashboard that consists of available resources and their status.

**Figure 22:**
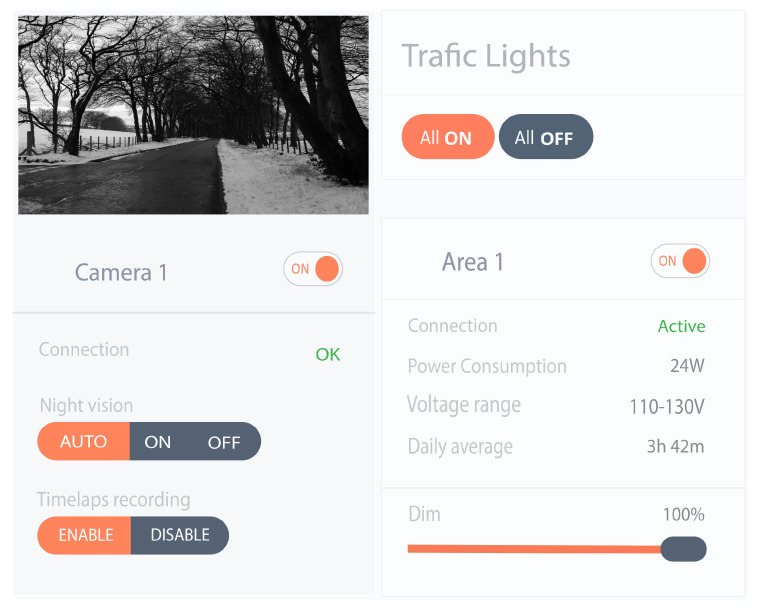
Resource description.

After the selection, the users can visualize and access the details related to the particular resource. Figure 22 shows the details of traffic lights located in Area 1 and information related to camera 1. The user can control these resources.

### 5.3. Comparative Analysis

In this work, a comparative analysis among proposed and existing techniques has been made on the basis of targeted domain, design, implementation, and user interface. Table 1 enlists the features supported by the proposed and existing solutions. It illustrates that most of the existing solutions do not incorporate all the essential features. Yue et al. [39] exploited Blockchain technology for the healthcare domain. Their work is comprehensive in nature comprising design, implementation, and user application. A Blockchain-based mechanism is presented in this work for secure authorization of smart city resources. It consists of the design and implementation of ACE framework-based authorization Blockchain and the OSCAR object security model. The Blockchain offers a flexible and trustless authorization mechanism. On the other hand, OSCAR makes use of a public ledger to establish multicast groups for authorized clients. Moreover, a meteor-based application is developed to provide a user-friendly interface for heterogeneous technologies belonging to the smart city to interact and control the smart city’s resources through this application.

### 5.4. Security Analysis

The secure authorized access to the IoT resources is the key to the proposed framework. Several aspects of security are being considered and implemented, as below.

#### 5.4.1. Formal Security Analysis

To formally analyze the security of the proposed system, authorization and authentication services provided by the proposed system have been evaluated as follows:

- Authorization: In the context of a Blockchain-based proposed mechanism, authorization means that an attacker should not be able to acquire or use encrypted resources accessible to some authorized clients $C^{n}$ at resource server $RS^{n}$ until more than 50% of nodes of authorization Blockchain $ABC^{n}$ are corrupted. Formally, it can be said that $ABC^{n}$ is secure with respect to authorization under the following conditions: if at a point an attacker can access the encrypted resources that are accessible to some authorized client $C^{n}$ at $RS^{n}$ for some resource owner $RO^{n}$, then the $RS^{n}$ is corrupt or, if $C^{n}$ ≠ ⊥, it implies $C^{n}$ or at least one of the trusted $RS^{n}$ of $C^{n}$ must be corrupted. As we know, if $C^{n}$ = ⊥, then the resource is accessed in the client credentials mode and is not related to a client.
- Authentication: In the context of a Blockchain-based proposed mechanism, authentication for resource server $RS^{n}$ means that an attacker should not be able to acquire an access token under the identity of a valid client $C^{n}$ unless certain parties involved are corrupted. Being authenticated at $RS^{n}$ means to have a valid access token from authorization server ($ABC^{n}$). Formally, it can be said that $ABC^{n}$ is secure with respect to authentication under the following conditions: if an attacker is able to access the service token issued by a valid $RS^{n}$ for a client $C^{n}$, then either the client $C^{n}$ or one of the valid resource servers $RS^{n}$ must be corrupted. In the case of the proposed Blockchain-based mechanism, the authentication and authorization are performed by authorization Blockchain $ABC^{n}$ nodes. Until more than 50% of nodes of $ABC^{n}$ are corrupted, the authorization can never fail. It is the realistic assumption adopted by the Blockchain that it would carry more honest nodes $H^{n}$ than the malicious nodes $M^{n}$. So, in our case, unauthorized access would not be possible because $H^{n}$ > $M^{n}$.
- Session Integrity: The session integrity from the authorization perspective is (1) a client $C^{n}$ is only authorized to access the resources of the resource server $RS^{n}$ when a client $C^{n}$ has a valid access token and (2) no other client $C^{n}$ can generate the access request on the behalf of the valid client $C^{n}$ because each access request is signed by the corresponding valid client $C^{n}$. Formally, it can be said that $ABC^{n}$ is secure with respect to session integrity for authorization if the following conditions are fulfilled: (1) if in an iteration for $ABC^{n}$, the challenge response flow is successful for a particular client $C^{n}$ and (2), additionally, the resource server $RS^{n}$ is honest to provide resources only to a client $C^{n}$ who already has successfully completed the challenge response flow. The authorization Blockchain $ABC^{n}$ is secure with respect to session integrity for authentication if the following conditions are fulfilled: (1) if in an iteration for $ABC^{n}$, the transaction request sent by a client $C^{n}$ is successfully verified by the miners and a valid access token is granted to it as a result of the execution of the smart contract and (2), additionally, the resource server $RS^{n}$ is honest to provide resources only to a client $C^{n}$ who already has successfully acquired the valid access token.

#### 5.4.2. Informal Security Analysis

To informally analyze the security of the proposed system, the following scenarios have been considered:

- Scenario-1: Personal keys may become compromised while sharing
  The personal keys are exchanged on DTLS channels which are based on TLS. So, there is no chance of eavesdropping, tampering, or message forgery while exchanging the keys. It ensures secure exchange of personal keys among different servers and clients. So, only the authorized client/server may obtain its key.
- Scenario-2: An unauthorized client may request a resource or a fake key server may be there
  Certificates are used to provide authentication between key and resource servers. The clients and key servers authenticate themselves through a challenge/response mechanism. Integrity of transactions are preserved by the use of signatures.
- Scenario-3: The access token may be captured and misused by the attacker
  The proposed system is built upon ACE. In ACE, the client identity is bounded with the access token using the concept of proof of possession (PoP). Whenever a request is sent from a client for the token, the ACE server binds a key to the token. The resource server can access this key and it is known to the client that placed the request. The resource server in turn creates a challenge response based on this key to verify whether the client is the owner of the token or not. So, what the attacker needs to do is to compromise only one authorization server to capture the valid tokens. In the proposed framework, this issue was resolved by demanding a public key from the client that triggers the smart contract. This public key of the client becomes part of the access token and is saved on Blockchain. The key server creates a challenge/response with the help of this key to verify the client’s identity who is demanding decryption keys. Now, an attacker is required to compromise at-least 51% of the miners to accomplish the desired task.
- Scenario-4: DoS attack can be performed on any network
  Denial of Service (DoS) attack possibility is always there and also on any infrastructure of Blockchain. Several measures are taken in the proposed framework to reduce the risk associated with such types of attacks. One possible scenario may be that a malicious user may repeatedly trigger smart contracts. This would create a huge volume of traffic in the Blockchain network as all requests need to be broadcasted and verified by the miners. It is minimized in a Blockchain-based network by presenting a client with some kind of a cryptographic puzzle before every transaction. This would flatten the attack. Furthermore, the execution time limit on the smart contracts is set on a lower side in the proposed framework. This helped in identifying the bogus requests without providing the gas value as is required in the Ethereum Blockchain. So, the smart contract would be halted in this case and all changes would be roll-backed.

### 5.5. Technical Challenges and Limitations

Following are the technical challenges for using Blockchain technology in a smart city environment:

- Both smart cities and Blockchain are in their infancy so a lot of research work is still required to integrate these.
- Shifting from a conventional centralized IoT system to a decentralized Blockchain network requires technical efforts to be made in right directions.
- Conventional security and privacy architectures are inapplicable in the spectrum of smart cities due to its resource constraints. Most of the IoT devices are not designed to meet the basic security and privacy parameters which produce the security, confidentiality, privacy, authentication, data integrity, and access control issues.
- A permissionless Blockchain is hard to implement and deploy in some institutions because it lacks in trust and is uncontrollable in nature.
- There are only a limited number of accounts available (i.e., 10 in the case of Ganache) to implement and test the efficacy of any proposed solution.

Although, Blockchain provides a decentralized security mechanism that helps to secure the authentication process, it has numerous limitations. The public Blockchain is hard to implement and deploy in government institutions due to its trustless and uncontrollable nature. All nodes in the ledger download and repeat the same data in mining at the same time which makes it more costly with respect to time and transactions. Every node takes part in the mining process which makes it slower as compared to the other centralized approaches. When any transaction is broadcasted, it will increase the size of the ledger at every node.

It is observed that public Blockchain in the proposed solution makes this framework less deployable due to speed, cost, complexity, and a bulk of data issues. However, it is useful for any type of pure private Blockchain such as multichain [62], hyperledger [63], chain core [64], etc. When the proposed architecture is developed on private Blockchain, then cost complexity, all node mining problems, and ledger size increment in the transaction can be controlled. Moreover, the speed issue can be reduced and solved with private Blockchain. There are many constrains that could also be applied to private Blockchain that may help to control unnecessary attachment of the user by blocking and controlling. Nodes can also be increased and decreased for the mining which can resolve speed issues. Private Blockchain such as multichain does not charge a cost for anything which solves the cost with respect to the execution cost such as satoshi in bitcoin [65], Ethereum gas [66], and eos [67]. The public Blockchains [65,66,67] have a cost factor that makes them less deployable, while some private Blockchains are open source and free [18]. It can be concluded that the proposed solution is more appropriate, fast, and secure for private Blockchain instead of public. Private Blockchain may also control area-wise access as well which makes it implementable by the government of any territory and state.

## 6. Conclusions and Future Directions

This paper presented a secure and reliable authentication and trust management mechanism for smart cities. The proposed mechanism is based on Blockchain technology. Moreover, a practical implementation of a Blockchain-based security mechanism for secure authorization of smart city resources is presented. Furthermore, a hybrid application is developed to provide a user-friendly interface for heterogeneous technologies belonging to the smart cities. The users would be able to interact and control the smart city’s devices such as traffic lights and surveillance with the help of this application. In the future, this work can be extended for healthcare, hospitality, medication, education, etc.

## Figures and Tables

**Table 1:** Features-based comparison of the proposed framework with existing solutions.

Techniques	Domain	Design	Implementation	User Interface
Yue et al. [39]	Blockchain in Healthcare	✓	✓	✓
Liang et al. [40]	Blockchain in Healthcare	✓	✗	✗
Jiang et al. [41]	Blockchain in Healthcare	✓	✓	✗
Fan et al. [42]	Blockchain in Healthcare	✓	✓	✗
Tanwar et al. [43]	Blockchain in Healthcare	✗	✗	✗
Habibzadeh et al. [44]	Cybersecurity in smart cities	✗	✗	✗
Khan et al. [45]	Blockchain for Smart cities	✓	✓	✗
Malik et al. [46]	Blockchain for Smart cities	✓	✓	✗
Meshram et al. [47]	Authentication in Smart cities	✓	✗	✗
Espositoa et al. [48]	Blockchain for Smart cities	✓	✓	✗
Proposed	Blockchain for Smart cities	✓	✓	✓

## Data Availability

Not applicable.
